# Pyonephrosis drained by double‐J catheter

**DOI:** 10.1002/ccr3.3204

**Published:** 2020-09-02

**Authors:** Che‐Wei Chang, Chun‐Nung Huang

**Affiliations:** ^1^ Department of Urology School of Medicine College of Medicine Kaohsiung Medical University Kaohsiung Taiwan; ^2^ Department of Urology Kaohsiung Medical University Hospital Kaohsiung Taiwan; ^3^ Department of Urology Kaohsiung Municipal Siaogang Hospital Kaohsiung Taiwan; ^4^ Department of Urology Kaohsiung Municipal Ta‐Tung Hospital Kaohsiung Taiwan

**Keywords:** double‐J catheter, hydronephrosis, pyonephrosis, ureteric stent

## Abstract

An infectious disease with obstructive hydronephrosis, pyonephrosis causes suppurative destruction of the renal parenchyma. In such cases, retrograde ureteric stenting is considered a good choice for the drainage of pyonephrosis, as an alternative to percutaneous nephrostomy and nephrectomy. The complication rate is minimal in retrograde ureteric stenting compared with about 4% in percutaneous nephrostomy.

## Graphical abstract

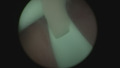

## 1 CASE SUMMARY

A 74‐year‐old woman presented to the emergent department with high fever and bilateral flank pain. Her medical history showed up cervical cancer, for which she had had radical hysterectomy, lymphadenectomy, and salpingo‐oophorectomy. Clinicians then performed regular double‐J catheter revision on the patient due to bilateral ureteral stricture.

**FIGURE 1:**
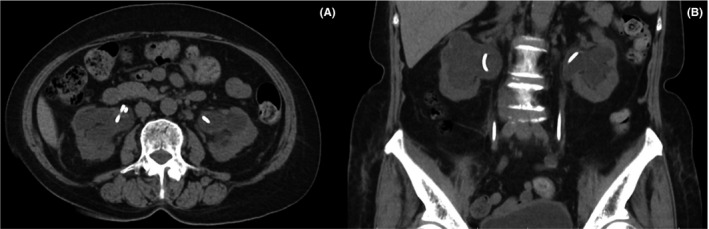
Abdomen computational tomography revealed bilateral pyonephrosis and hydronephrosis with previous double‐J catheters. A, axial view and (B) coronal view

**FIGURE 2:**
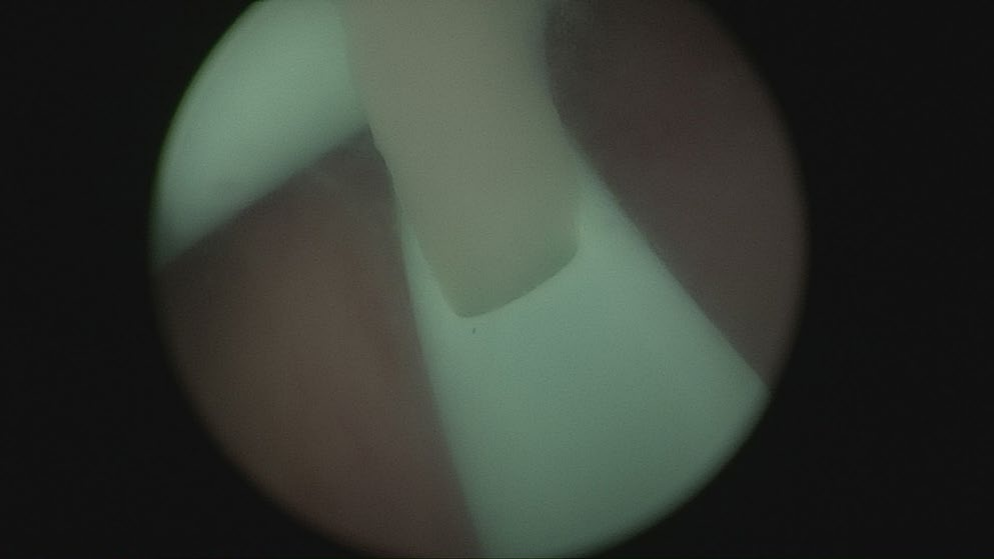
Pus‐like material drained from a side hole of right double‐J catheter, confirming the diagnosis of pyonephrosis

A subsequent examination went on to reveal tenderness on tapping the costovertebral angle. Urinalysis revealed pyuria, leukocytosis (white‐cell count of 16 650 mm^3^), elevated C‐reactive protein levels (212.2 mg/L), hyperkalemia (5.2 mmol/L), and deteriorated renal function (eGFR:12.61 mL/min/1.73 m^2^). Computational tomography of the abdomen showed persistent bilateral hydronephrosis (Figure 1). Cystoscopy revealed turbid urine because the right double‐J catheter, placed 3 months previously, had been dislodged. On this basis, bilateral double‐J stenting was performed to revise the last. Following this, pus‐like material drained from a side hole of the double‐J stent, confirming the diagnosis of pyonephrosis (Figure 2, Video S1). Continuous bladder irrigation was done, along with the drainage of the double‐J catheter. The patient's condition gradually improved, until she was discharged.

Pyonephrosis is an infectious disease usually accompanied by obstructive hydronephrosis, which causes the suppurative destruction of the renal parenchyma. Retrograde ureteric stenting is a good choice for the drainage of pyonephrosis, as an alternative to percutaneous nephrostomy and nephrectomy.^1^ Both retrograde ureteric stenting and percutaneous nephrostomy are comparably effective in the decompression and resolution of sepsis. The complication rate of retrograde ureteric stenting is minimal, but for percutaneous nephrostomy, it is about 4%.^2^

## CONFLICT OF INTEREST

The authors declare no conflicts of interest.

## AUTHOR CONTRIBUTIONS

CNH: performed the surgery. CWC: reviewed this case and was a major contributor in writing the manuscript. All authors read and approved the final manuscript.

## ETHICS APPROVAL AND CONSENT TO PARTICIPATE

Approval for the study was obtained from the institutional review board of Kaohsiung Municipal Ta‐Tung Hospital.

## CONSENT FOR PUBLICATION

Informed consent was obtained from the patient for the publication of this case report.

## Supporting information

Video S1

## Data Availability

The authors do not wish to share the patient's data. The privacy of this participant should be protected.
